# Thermodynamics Properties of Leucine and Isoleucine Peptides in Water

**DOI:** 10.1002/open.202400209

**Published:** 2025-01-28

**Authors:** Samundra Chapagain, Shishir Ojha, Shyam Prakash Khanal, Narayan Prasad Adhikari

**Affiliations:** ^1^ Central Department of Physics Tribhuvan University Kathmandu Nepal

**Keywords:** Molecular dynamics simulation, Free energy of solvation, Thermodynamic integration

## Abstract

Thermodynamic properties of amino acids explore the ideas about the energetic contribution in biomolecular interfaces. In our work, we have estimated the solvation free energy of leucine and isoleucine peptides with the variation of chain length or residues of different monomer units (n=1, 2, 4, 8 & 16) using molecular dynamic simulation. We modeled our system using OPLS‐AA force field and TIP3P water model at 310 K temperature. Solvation free energy of both leucine and isoleucine peptides increases with increase in chain length, which have been reported by using TI, TI‐CUBIC and BAR methods. The increase in solvation free energy with increase in chain length of both peptides is also supported by the increase in hydrogen bond and solvent accessible surface area (SASA) with the number of residues.

## Graphical abstract

This article focuses on the thermodynamic properties of leucine and isoleucine peptides in water focusing on solvation free energy, SASA, and hydrogen bonds for TIP3P water model at 310 K temperature. This provides new insights into the interaction of these amino acids in aqueous environments, highlighting key factors influencing their stability and molecular characteristics. It is seen that the solvation free energy increases with increase in chain length of the peptide.

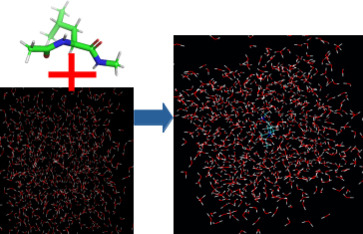

## 1 Introduction

Amino acids are considered as basic structural and fundamental building block of protein that differs from other biomolecules such as carbohydrates and lipids, as they are not stored in our body. They can supplied in our body through different forms such as dietary proteins, proteolysis of tissue proteins and biosynthesis of non‐essential amino acids, tissue proteins, functional proteins; non‐protein nitrogenous substances [1]. For the formation of polymer chains different amino acids are joined together which are called as peptides that are formed between carboxyl group of one amino acids and amino group of another amino acids. The series of a chain of amino acid residues in a linear pattern are poly peptides whereas the proteins are macromolecular polypeptides. Polypeptides contain the aliphatic chain as well as the carboxyl and amino group. For the discovery of new drug, life sciences, archaeological, nutrition, food science, human physiology, geological specimen poly peptide play an pivotal role [2].

Among the various amino acids; we are studying about the leucine and isoleucine. Leucine and isoleucine are the structural isomers that possess branched chains and proteinogenic essential amino acids which contains the same carboxyl and amino functional groups but differs only on the arrangement of the carbon atoms [3]. As they have different structures they bear different functions. They bears the common function such as the regulation of blood sugar level from gluconeogenesis; metabolism process such as catabolism and anabolism so as to construct the components of biomolecules carbohydrates; nucleotides; amino acids etc and assembled to complex molecules and breaks down the complex molecules into carbohydrates, fat and protein. Stress responses such as immediate energy to flight or fight situation such as high blood pressure, heart disease and an anxiety disorders. For the process of electron affinity; there exists two transport properties i. e. high electron affinity and low electron affinity. High electron affinity is seen in both the leucine and isoleucine [4].

Solvation free energy is an interaction of cation and anion in gaseous phase with a solvent in liquid phase at a certain temperature and pressure that leads to a free energy change. It is sudden, immediate and random change of thermodynamic parameters. Both implicit and explicit water models are used for the estimation of solvation free energy for the complex biomolecular system [5]. It arises due to the intermolecular attraction and intramolecular attraction. These processes are performed by the theoretical and computational techniques to elaborate and explore the knowledge to obtain the various thermodynamic parameters. Solvation free energy is more tractable than binding free energy, since the solvent molecules equilibrium more quickly around a small organic solute than around the binding size of the protein. It incorporates the important bio‐chemical and physical processes such as association, dissociation, binding constant and energy, fold on energy, partition constant, absorption coefficient, diffusion, solubility etc [6]. Though there are the senses of the method for recalculation of solvation free energy, we would like to under go the thermodynamic integration and free energy perturbation for calculating the solvation free energy of the branched amino acids (leucine and isoleucine).

In the liquid phase, molecular dynamics simulations is used to analyze and calculate Hydrogen bonding, solvation free energy, viscosity, densities, etc [7, 8]. We calculate solvation free energy of amino acid peptides derived from the OPLS‐AA parameter set. Different models are explored for the calculation of solvation free energy but we are heading towards the best suited model i. e. TIP3P water model. This model is a model designed to give improved free energy of solvation which gives uniformly closest match to experiment [9, 10].

**Figure 1:**
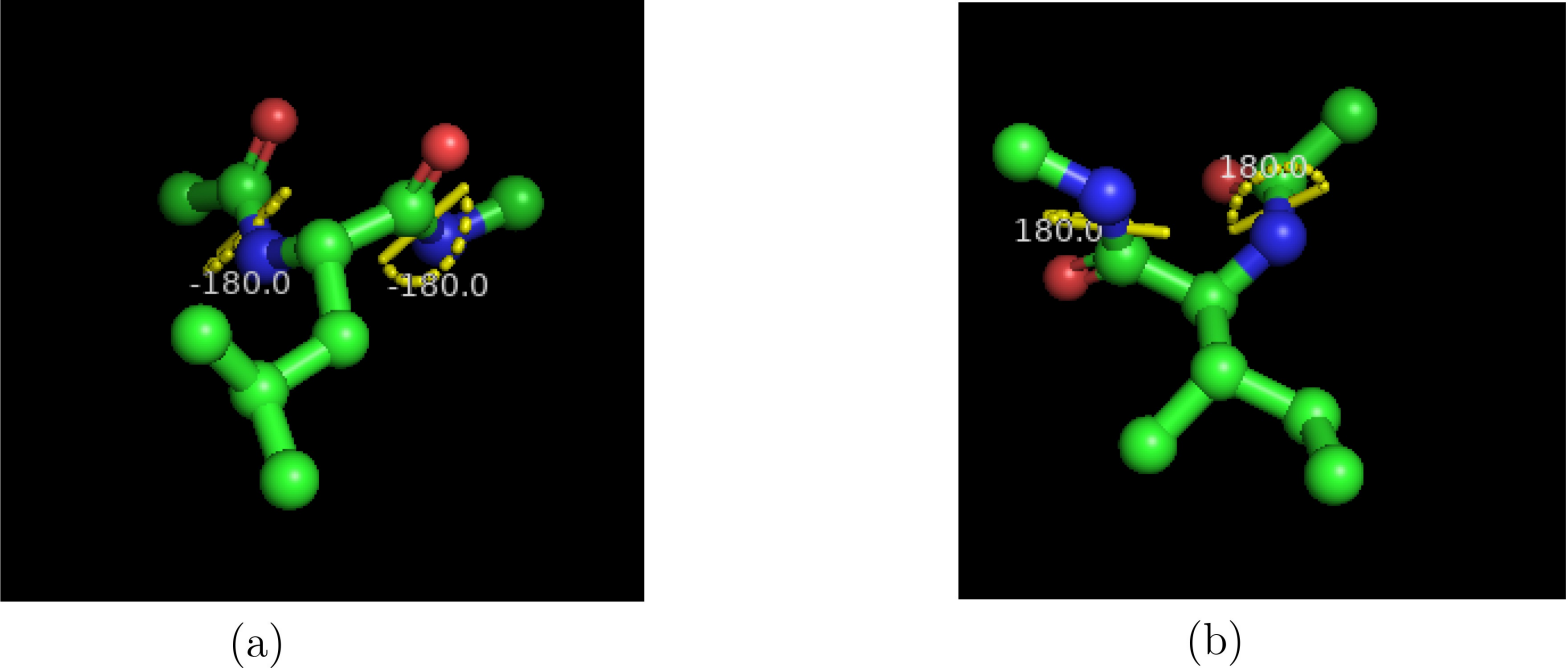
Molecular structure of capped monomer (a), leucine peptide (b), isoleucine peptide using Pymol.

With the aid of Molecular Dynamics (MD) simulation, we have studied the solvation free energy of leucine and isoleucine peptides with different chain length in water at 310 K temperature. Figure 1 represents the molecular structure of capped leucine and isoleucine monomer. They are considered as the structural isomers that is the reason why their physiological properties are changed. Both leucine and isoleucine are called the branched chain amino acids. They are also called as the isobaric amino acids as both have the same mass.

## Methods and Methodology

The reliability and compactibility depends on the adaptation of the technique as the series of the method via molecular dynamics simulation formulated are excellent. During this work, we work using alchemical free energy difference with the thermodynamic state points not only in the configuration but consider the whole phase space. The free energy difference which are calculated basically depends upon three aspects such as selection of model, selection of sampling protocol and selection of estimator of free energy difference [11]. The selection of the model means the selection of the Hamiltonian to calculate the various factors such as the energy and forces in accurate and efficient way. The selection of the sampling protocols gives the information about the ensembles and partition function. The selection of estimator of free energy difference is done by Thermodynamic Integration (TI) in our work. As mentioned earlier it considers the whole phase but it is not only restricted in these matters but also prepares the framework that how the solvent molecules behaves in the different aqueous environment. In this work, as the solvent molecules behaves in water so it also be referred as the hydration free energy. According to the simulation protocol, solvation free energy is the sum of the electrostatic interaction and van der Waals interaction in case of non‐bonded interaction Kokubo [12].

(1) $$\Delta G_{Total}=\Delta G_{Coulomb}+\Delta G_{vdW}$$

The electrostatic components and van der Waals components related to the Lenard Jones potential and Coulomb potential respectively. In order to calculate the free energy difference between one thermodynamic state to another thermodynamic state accurately and suitably, TI is choosen [13]. It uses the various intermediate states in which the system changes from one form to another. The generalized form of integration method where an adiabatic switching is used and it provides a fruitful environment at each and every point of the various intermediate states. In thermodynamic state, there is no compulsion for overlapping of intermediate states in phase space [14].

According to the TI approach, the free energy difference between two states A and B can be calculated by using relation [15].

(2) $$\Delta G_{AB}=\int_{0}^{1}{\left\langle \frac{\partial U}{\partial \lambda }\right\rangle }_{\lambda }d\lambda$$

${\left\langle ...\right\rangle }_{\lambda }$ represents the ensemble average at a particular value of λ . λ is a coupling parameter which ranges from 0 to 1 with the series of intermediate states according to the Hamiltonian $H_{0}$ and $H_{1}$ respectively. Various values of the coupling parameter combined by the numerical integration formulas, the main problem of time consuming for this method as it contains a lot of non‐physical intermediate states. The non‐physical intermediate states itself is not a drawback but appears as a gaining of computational works. Various approaches are available for the numerical integration of thermodynamic integration in the frames of alchemical transformation. In our work, we carry out TI‐1 uses trapezoidal rule (first order polynomial) and TI‐3 uses cubic spline ($3^{rd}$ order polynomial) that only differs only on the interpretation of our results obtained.

A protocol based on a simple, classical statistical mechanics for calculating the free energy difference between two states of a system is free energy perturbation (FEP) method. Solvation free energy is the interaction of gaseous and liquid phases. By using FEP, we can estimate the free energy difference between two states A and B as [15];

(3) $$\Delta A_{AB}=-k_{B}T\ln\left\langle \exp\left\{-\beta [U_{B}-U_{A}]\right\}\right\rangle$$

where the notation ⟨...⟩ indicates an average taken with respect to canonical configurational distribution of the state A and $U_{A}$ and $U_{B}$ are potential energies with two states A and B, $k_{B}$ is the Boltzmann constant, T is temperature and $\beta =\frac{1}{k_{B}t}$ .

### Simulation Details

As both experiment and theory are compared using a simulation there is no debate to consider it as a bridge between them. Various techniques are used in the computer simulation but here we deals only with the molecular dynamics simulation. System preparation is considered as the initial step for performing the calculation via simulation. First of all we are proceeding towards making the structure preparation of leucine and isoleucine peptides. After this we have now PDB structure of leucine and isoleucine peptide that is considered as initial step of system preparation. Once we get a PDB structure we need to check it that their entries may contain the unnecessary atoms or missing atoms as it creates a error or problem. The PDB structure is inserted into the gromacs file i. e. pdb2gmx. It consists of three different files i. e. topology file, position restrain file and post‐processed structure file using OPLS‐AA force field [16‐18]. After the generation of topology file; next step is to define the box dimensions and fill the box with water using solvent molecule. Water is a natural solvent for both leucine and isoleucine peptides. For this we use a simple cubic box as the unit cell using the command gmx editconf, solvent is placed at the centre of box and placed it at a distance of 1.0 nm from box edge. Periodic boundary condition is needed for water box edge. The calculation of the forces would be factitious if it is seen it's periodic image. The distance between two periodic images of protein must be at least 2.0 nm to specify the solute box distance that is sufficient for cutting off scheme in simulations.

Water is considered as a natural solvent for the solvation mechanism. The simple cubic box is considered as a unit cell where solvent is placed at the centre of the box and is at a distance of 1.0 nm from the box edge. Also, the periodic boundary conditions are needed for the water box creation. To specify the solute box distance that is sufficient for cutting off scheme in simulations. The distance between two periodic images of protein must be at least 2.0 nm.

Energy minimization starts when we fill the box with water into solvate module. It helps to adjust the structure of force field and distribution of solvent molecules as well as minimization of unnatural overlapping of the atoms. We are heading towards the Steepest Decent Algorithm [16, 19] which terminates when maximum of the absolute values of the force components is less than specified value. The existence of the equilibrium is determined by the various thermodynamic parameters such as density, temperature, pressure and other variables that are distributed through out the system with in a specific period of time [20, 21]. Our system is equilibrated on two thermodynamic ensembles NVT and NPT for 1 ns with steps 1250000 using 2 fs time step. NVT is carried out at a temperature of 310 K by means of Langevins thermostat with coupling time 1 ps and NPT is carried out at a pressure of 1 bar by mean of Parinello‐Rahman barostat with coupling time 1 ps. After equilibration of thermodynamic parameter; the simulation move towards the production run for 2500000 steps with 0.002 ps. Production run is carried out by using NVT ensemble only [22]. LINCS algorithm is used for the constrants of hydrogen bonds [23]. As the potential is a function of two parameters i. e. $\lambda_{C}$ for electrostatic interaction and $\lambda_{LJ}$ for van der Waals interaction as: $\lambda_{C}=$ 0.00, 0.00, 0.00, 0.00, 0.00, 0.00, 0.00, 0.00, 0.00, 0.00, 0.00, 0.10, 0.20, 0.30, 0.40, 0.50, 0.60, 0.70, 0.80, 0.90 and 1.00 and $\lambda_{LJ}=$ 0.00, 0.10, 0.20, 0.30, 0.40, 0.50, 0.60, 0.70, 0.80, 0.90, 1.00, 1.00, 1.00, 1.00, 1.00, 1.00, 1.00, 1.00, 1.00 and 1.00. For each λ state, production run has been carried out under NPT ensemble to estimate solvation free energy of both peptides using alchemical‐analysis.py [23, 24].

## 2 Results and Discussion

In this section, we have presented the details outcomes elicited from solvation free energies of the leucine and isoleucine peptides with chain length n=1,2,4,8, and 16 in water at 310 K temperature. We compare the computed solvation free energies with different methods (TI, TI‐CUBIC and BAR).

### 2.1 Free Energy of Solvation

Solvation free energy of leucine and isoleucine peptides are calculated using FEP and TI based method. Thermodynamic cycles are constructed in order to derive the solvation free energy of isoleucine and leucine in water. As water is polar in nature, it consists of the electrostatic interactions and van der Waals interaction. Solvation thermodynamics is constructed with the fixed number of atoms; having a same volume and fixed temperature (310 K) i.e. NVT ensemble.

We work using alchemical free energy difference with the thermodynamics state point that concerns with the thermodynamic state points not only in the configuration but also considers the whole phase space as we deal with the molecular dynamics simulation. Each peptides of leucine and isoleucine are capped with acetyl (−ACE) and N‐methylamine (−NME) at C‐ and N‐ terminus respectively. Capping acts as a remedy or weapon of treatment of impurities in the structure of peptides and blocked the N‐ and C‐ terminus at the unreacted sites. As both consists of amine group i.e $NH_{2}$ and carboxyl group i. e. COOH, it appears in the form of zwitterion structure.

Solvation free energies are estimated by the transferring of leucine and isoleucine peptides from the ideal condition to solvent at a certain limit of the temperature and pressure. To study the performance of leucine and isoleucine peptides we here adopted the TI and TI‐CUBIC methods. We have estimated the change in free energy ΔG that integrates the ensemble average of $\frac{\partial U}{\partial \lambda }$ with respect to the different λ ‐states from 0 to 1 instead of calculating the free energy between subsequent states. The effects of electrostatic and van der Waals interaction are noticed with the conditions of the on and off surting of λ ‐states.

**Figure 2:**
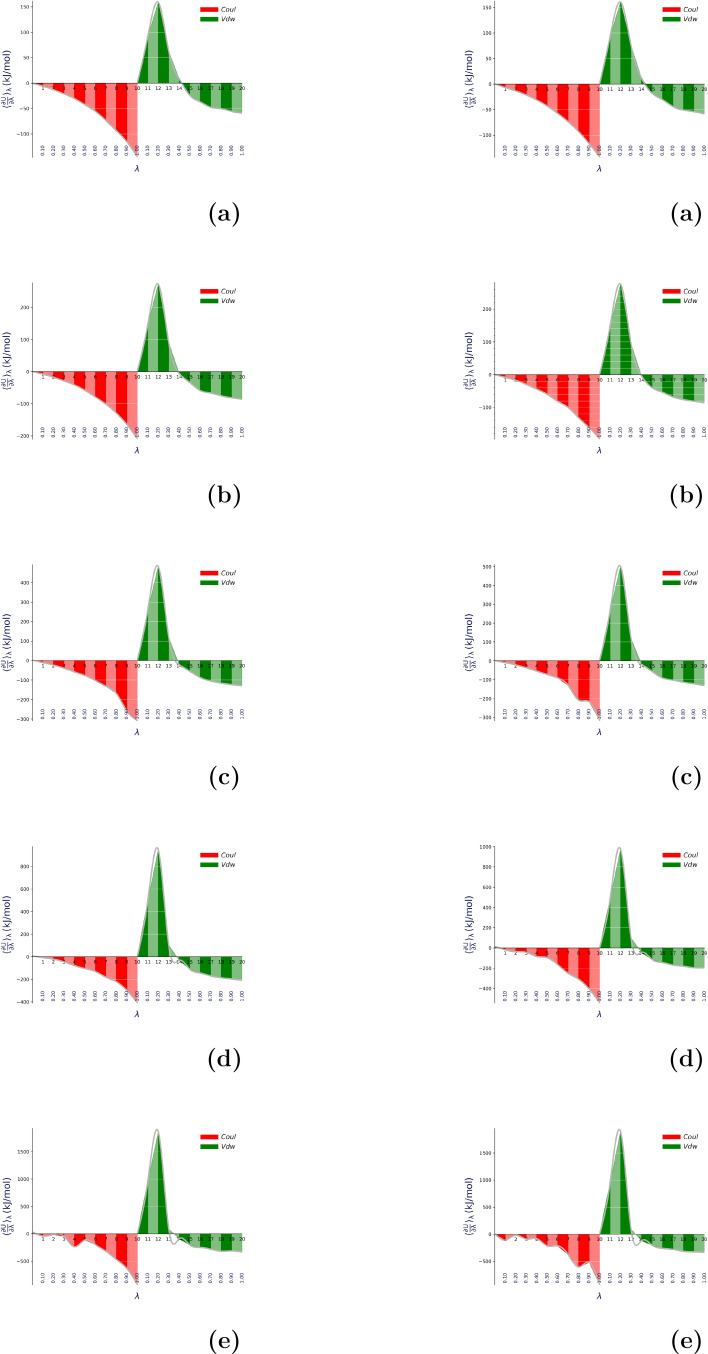
Variation of ${\left\langle \frac{\partial U}{\partial \lambda }\right\rangle }_{\lambda }$ as a function of λ at 310 K temperature taking TIP3P water model for ACE‐L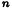 ‐NME (left) and ACE‐I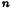 ‐NME (right) with n=1 (a), 2 (b), 4 (c), 8 (d), and 16 (e).

**Figure 3:**
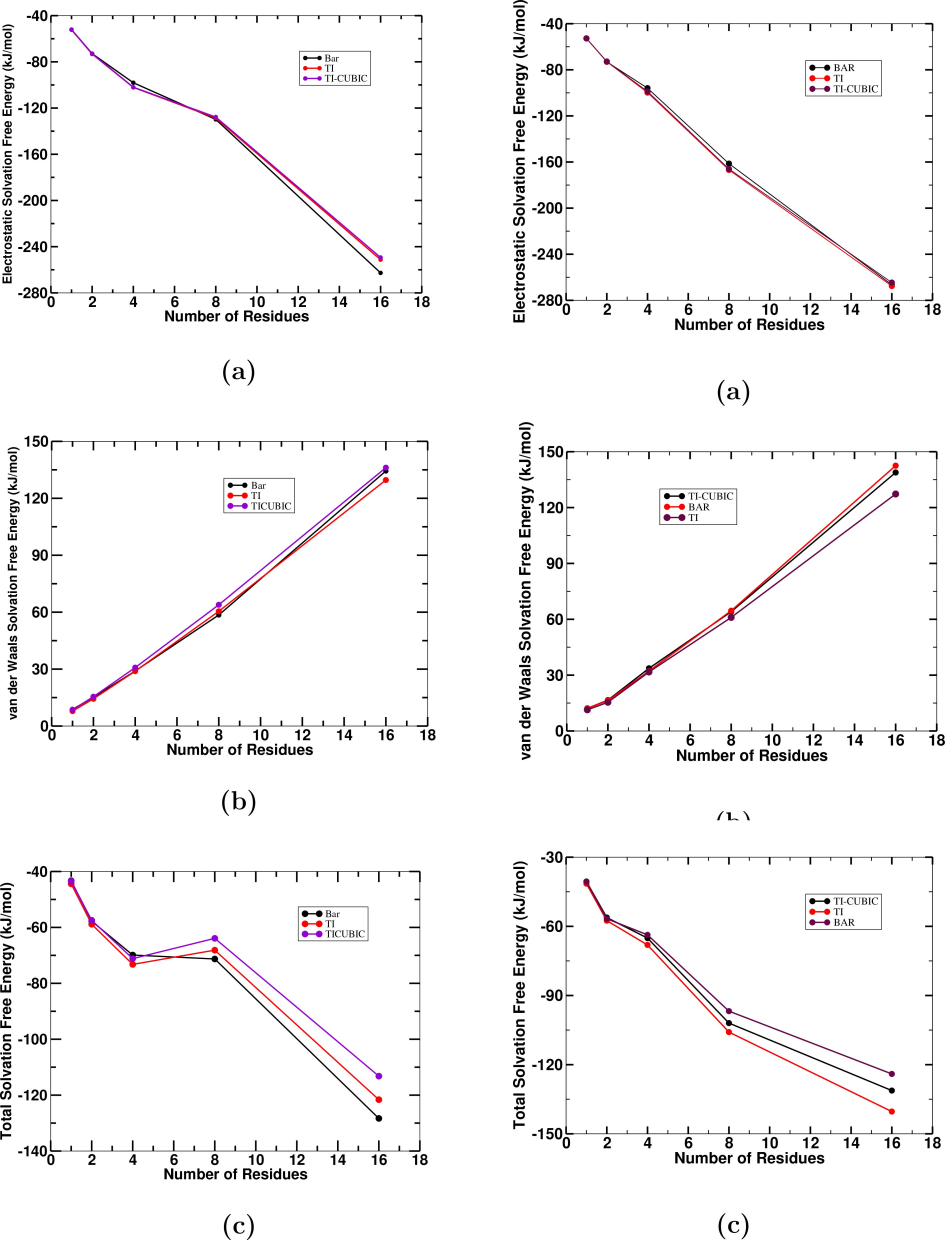
Solvation free energies: (a) Coulombic solvation free energy, (b) van der Waals solvation free energy, and (c) total solvation free energy of leucine (left) and isoleucine (right) peptides as a function of number (n) of residues or monomers in the peptide, using BAR, TI and TI‐CUBIC.

In Figure 2 we noticed the filled area (Red and Green) estimates the free energy from trapezoidal rule i. e. first order polynomial and the silver curve represents the interpolation i.e cubic spline (third order polynomial). First order polynomial is TI and third order polynomial is TI‐CUBIC. From the plotting, it can be easily observed that at the initial state, the change in free energy is zero which means it is in the final state. Then, the electrostatic component goes on increasing constantly and continuously but with the negative sign. The negative sign indicates that the interaction involved in solvation and self‐solvation is exothermic. When an electrostatic interaction becomes maximum then it is switched off and the van der Waals interaction starts into it's effect. The van der Waals interaction shows it‘s positive effect from λ=0.1 to 0.4; neither positive nor negative at λ=0.5 and shows the negative effect from λ=0.6 to 1. The electrostatic contribution dominates the van der Waals contribution due to the presence of both positive and negative contribution in van der Waals. The effective van der Waals interaction starts after the complex break down of electrostatic interaction. In other words; at λ=10 states onwards; there is no effect of electrostatic interaction and effects of van der Waals interaction starts. Both the contribution increases continuously as the number of residues increases. Figure 3 illustrates the individual comparision of the electrostatic and van der Waals components and as a whole of the total solvation of the leucine and isoleucine peptides from TI and TI‐CUBIC methods.

The simulation results for the solvation free energies of leucine and isoleucine peptides in water at 310 K using different methods (TI, TI‐CUBIC and BAR) are mentioned in Table 1. We observed that the van der Waals solvation free energies are positive and have small contribution to the total solvation free energy for leucine and isoleucine peptides. However, strongly negative coulombic contributions have a significant impact on the total solvation free energies for both peptides. The increase in negative values of coulombic contribution with the increase in chain length is due to the fact that adding monomers introduces a considerable portion of peptide dipole to a polar solvent. For both peptides, the negative values of total solvation free energy increases with increase in chain length. These results also gives the evidence that the results are in good agreement or they are nearly consistent with each other. Also, we noted that the significant errors for the calculation of solvation free energy is less.

**Table 1:** Estimated values of solvation free energy ($\Delta G_{vdW}$ , $\Delta G_{Coulomb}$ , $\Delta G_{Total}$ ) of leucine and isoleucine peptides of monomers n=1, 2, 4, 8, and 16 in water at 310 K using TI, TI‐CUBIC and BAR methods.

Peptide	n	Method	$\Delta G_{sol}$ in kJ/mol with		
			vdW only	Coulomb only	Total
Leucine	1	TI	7.9 ± 0.3	−52.3 ± 0.1	−44.4 ± 0.3
		TI‐CUBIC	8.6 ± 0.2	−52.0 ± 0.1	−43.4 ± 0.3
		BAR	8.8 ± 0.2	−52.0 ± 0.1	−43.2 ± 0.2
	2	TI	14.3 ± 0.3	−73.2 ± 0.2	−58.9 ± 0.4
		TI‐CUBIC	15.5 ± 0.3	−73.0 ± 0.2	−57.5 ± 0.4
		BAR	15.1 ± 0.4	−72.9 ± 0.2	−57.8 ± 0.4
	4	TI	28.9 ± 0.4	−102.1 ± 0.5	−73.2 ± 0.7
		TI‐CUBIC	30.8 ± 0.4	−102.0 ± 0.6	−71.2 ± 0.7
		BAR	29.0 ± 1.2	−98.9 ± 0.3	−69.9 ± 1.2
	8	TI	60.5 ± 0.6	−128.6 ± 0.7	−68.1 ± 1.0
		TI‐CUBIC	63.9 ± 0.6	−127.8 ± 0.7	−63.9 ± 1.0
		BAR	58.5 ± 3.6	−129.8 ± 0.4	−71.3 ± 3.6
	16	TI	129.6 ± 0.9	−251.2 ± 3.0	−121.6 ± 3.2
		TI‐CUBIC	136.1 ± 0.9	−249.4 ± 3.1	−113.3 ± 3.2
		BAR	134.4 ± 2.3	−262.7 ± 0.8	−128.3 ± 2.5
Isoleucine	1	TI	11.4 ± 0.1	−52.9 ± 0.1	−41.5 ± 0.1
		TI‐CUBIC	12.2 ± 0.1	−52.7 ± 0.1	−40.5 ± 0.1
		BAR	12.2 ± 0.2	−52.7 ± 0.1	−40.5 ± 0.3
	2	TI	15.4 ± 0.2	−73.0 ± 0.3	−57.6 ± 0.4
		TI‐CUBIC	16.6 ± 0.2	−72.8 ± 0.4	−56.2 ± 0.4
		BAR	16.4 ± 0.2	−73.0 ± 0.1	−56.6 ± 0.3
	4	TI	31.7 ± 0.3	−99.8 ± 0.2	−68.1 ± 0.4
		TI‐CUBIC	33.7 ± 0.3	−98.8 ± 0.2	−65.1 ± 0.4
		BAR	32.2 ± 0.2	−96.0 ± 0.1	−63.8 ± 0.3
	8	TI	61.0 ± 0.5	−166.9 ± 1.2	−105.9 ± 1.3
		TI‐CUBIC	64.0 ± 0.5	−166.0 ± 1.3	−102.0 ± 1.4
		BAR	64.6 ± 0.2	−161.3 ± 0.1	−96.7 ± 0.3
	16	TI	s 127.3 ± 2.6	−267.6 ± 3.6	−140.3 ± 4.4
		TI‐CUBIC	133.3 ± 2.5	−264.6 ± 3.5	−131.3 ± 4.3
		BAR	142.5 ± 0.2	−266.5 ± 0.1	−124.0 ± 0.3

Figure 3 represents the comparison between the total solvation free energy, coulombic interaction and van der Waals interaction of leucine and isoleucine peptides obtained from TI, TI‐CUBIC and BAR methods. From this graph, we can observe that their nature are almost consistent with each other as they are nearly identical and good agreement with each other. From the nature of the total solvation free energy of both leucine and isoleucine peptides; the major contribution is of Coulombic as their graphical format are more or less similar to that of the total solvation free energy. In case of total solvation free energy they possess linear behaviour with the n≥8 .

### 2.2 Hydrogen Bonds and Solvent Accessible Surface Area (SASA)

To study the protein structure and their motion requires a knowledge of hydrogen bonds and their relative strengths [25]. Hydrogen bonds are an attractive intermolecular force that affects many physical properties of matter such as boiling point, vapour pressure, state of matter and solubility, etc. In this study, the hydrogen bonds between solute and solvent were examined for leucine and isoleucine peptides. During the hydrogen bond analsis, the cutoff parameters for length and angle are 0.35 nm and $30^{\circ }$ respectively.

**Figure 4:**
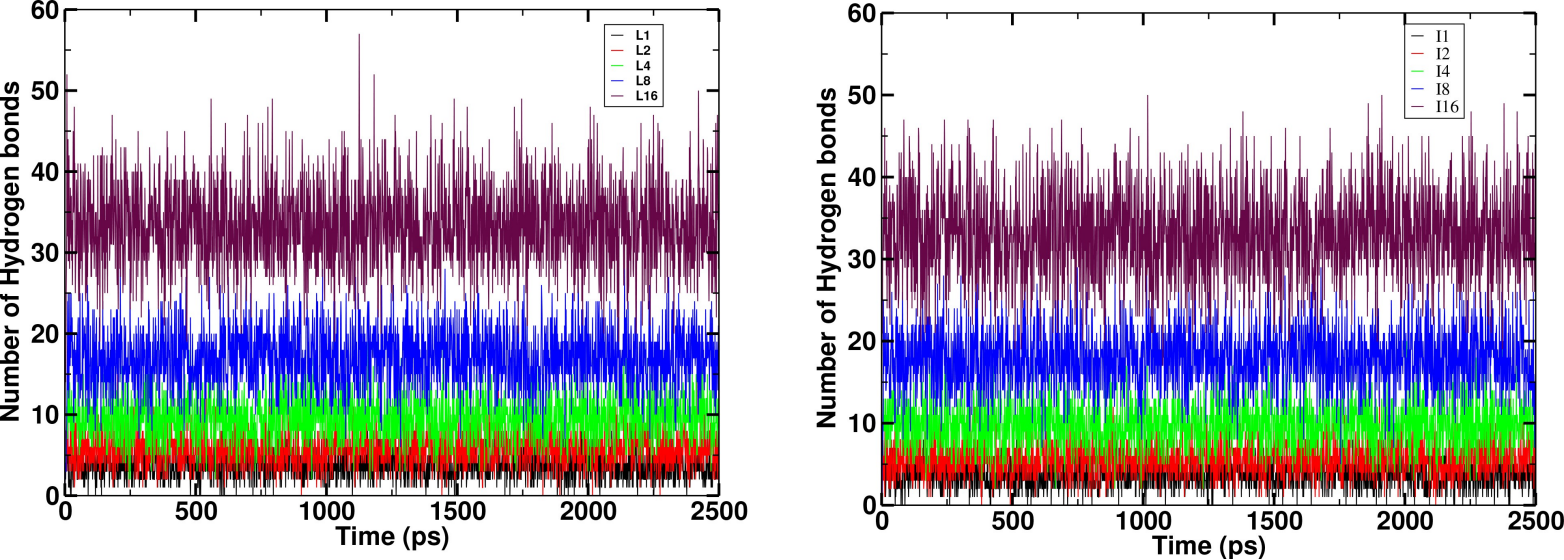
Comparison of time evolution of number of hydrogen bonds between leucine peptides and water at 310 K temperature for coupling state λ =1.

**Figure 5:**
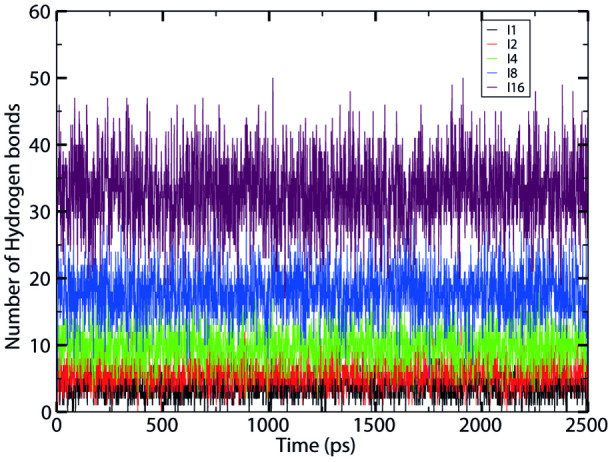
Comparison of time evolution of number of hydrogen bonds between isoleucine peptides and water at 310 K temperature for coupling state λ =1.

Figures 4 and Figure 5 show the time evolution of hydrogen bonds formed during interaction of leucine and isoleucine peptides respectively with water. There are approximately 3, 5, 8, 16, and 35 hydrogen bonds for the leucine peptides with chain length n=1,2,4,8 and 16 and 3, 6, 10, 18, and 36 hydrogen bonds for isoleucine peptides with chain length n=1,2,4,8 and 16 respectively. This indicates that the number of hydrogen bonds between solute and solvent increases with increase in chain length. We can conclude that as the chain length increases, there is an increase in interaction between peptides and water as expected. When we compare the hydrogen bonds between leucine and isoleucine peptides, Isoleucine has sec‐butyl side that allows for the more explorable orientations and interactions, rather than that of more branched isobutyl side chain of leucine. Moreover, the peptide conformation and steric hindrance limits the formation of H‐bonds. Though both of them are the non‐polar but in comparison isoleucine are slightly more polar than that of leucine [8].

The surface area of the biomolecules such as lipids, proteins and amino acids with their derivatives that are convenient and easily approachable to the solvent [10, 25]. Within the framework of free transfer energy different solvents environments are made focused in it as an indicator or parameters of solute solvent interactions. In our case, the solvent is referred as water. SASA is the sketching and representation of the surface around a protein contact with the functional groups [26]. The two techniques used in free energy of folding of protein are the totaling of the contributions of the intermolecular interactions and interactions of the molecules with surrounding solvent.

**Figure 6:**
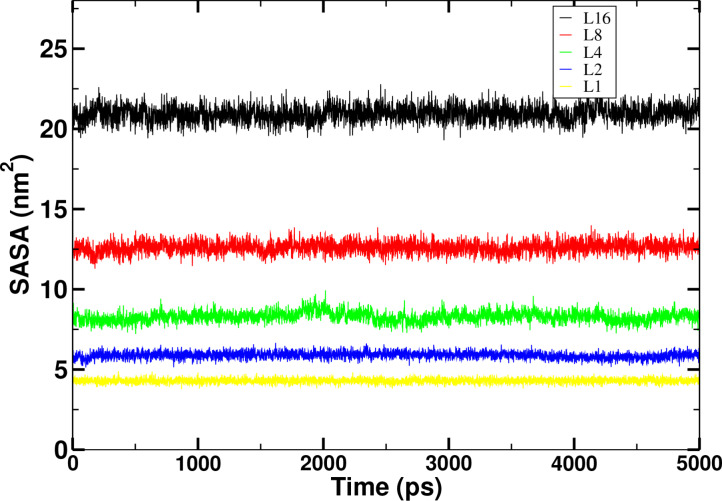
Comparison of time evolution of Solvent Accessible Surface Area (SASA) of leucine peptides at 310 K temperature for coupling state λ =1 in aqueous environment.

**Figure 7:**
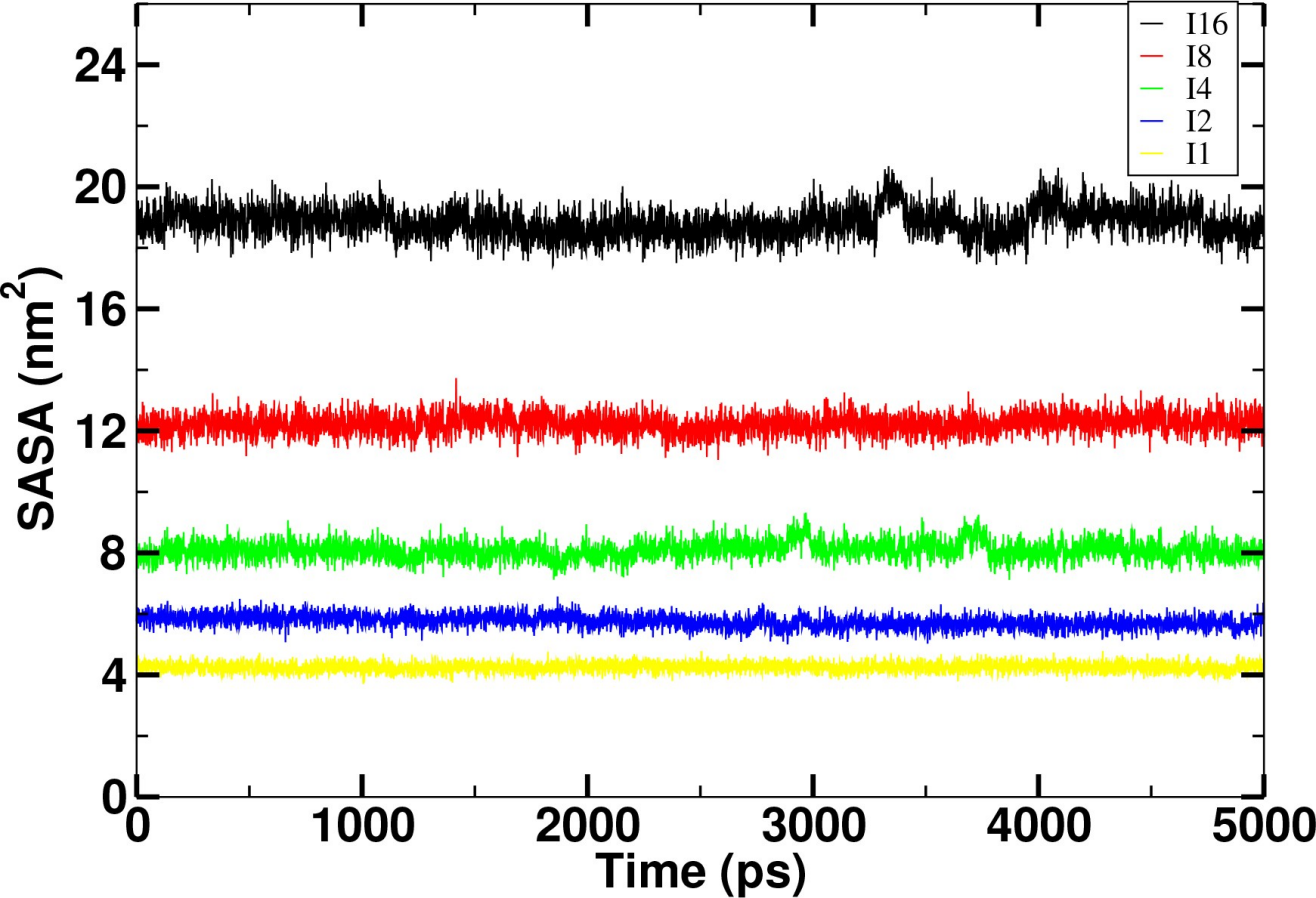
Comparison of time evolution of Solvent Accessible Surface Area (SASA) of isoleucine peptides at 310 K temperature for coupling state λ =1 in aqueous environment.

Figure 6 and Figure 7 show that the SASA values in case of leucine and isoleucine peptides respectively. With the increase in the chain length, the SASA values also increases. With this increment with the increase in the number of residues, it is also directly related to the solvation free energy. It also gives us an information about the configuration change as it is absorbed in each increases in chain length in both leucine and isoleucine peptides. It plays an immense and pivotal role in the folding and stability of the proteins for the estimation and prediction of the various thermodynamic parameters [27].

## 3 Conclusions and Concluding Remarks

With the aid of molecular dynamic simulations, we have been estimated the solvation free energies of leucine and isoleucine peptides with chain length n=1,2,4,8, and 16 in water at 310 K temperature. For the modeling of our system, TIP3P water model was used with OPLS/AA force field during the simulation. We have estimated the solvation free energy of leucine and isoleucine peptides using TI, TI‐CUBIC and BAR methods. It is observed that the van der Waals interaction are positive and small contribution to the total solvation free energy where as Coulombic interaction has negative and significant contribution to the total solvation free energy. The negative value of total solvation free energy increases with increase in chain length for both leucine and isoleucine. Here comparisions are made for all of the outcomes obtained from simulations using different methods(TI, TI‐CUBIC and BAR). We also observed that the number of hydrogen bonds between peptides and water as well as SASA increases with increase in chain length of the peptides. This research work can be further extended by increasing the chain length of the peptides and taking other solvents.

## Conflict of Interests

The authors declare no conflict of interest.

4

## Data Availability

All the data that are used to produce the Figures/Tables in this paper are available from the corresponding author in case reproduction is needed.
